# Magnetic hyperthermia therapy in glioblastoma tumor on-a-Chip model

**DOI:** 10.31744/einstein_journal/2020AO4954

**Published:** 2019-12-20

**Authors:** Javier Bustamante Mamani, Bruna Souto Marinho, Gabriel Nery de Albuquerque Rego, Mariana Penteado Nucci, Fernando Alvieri, Ricardo Silva dos Santos, João Victor Matias Ferreira, Fernando Anselmo de Oliveira, Lionel Fernel Gamarra

**Affiliations:** 1 Hospital Israelita Albert Einstein, São Paulo, SP, Brazil.; 2 Hospital das Clínicas, Faculdade de Medicina, Universidade de São Paulo, São Paulo, SP, Brazil.

**Keywords:** Glioblastoma/therapy, Hyperthermia, Nanoparticles, Microfluidics, C6 cells

## Abstract

**Objective:**

To evaluate the magnetic hyperthermia therapy in glioblastoma tumor-on-a-Chip model using a microfluidics device.

**Methods:**

The magnetic nanoparticles coated with aminosilane were used for the therapy of magnetic hyperthermia, being evaluated the specific absorption rate of the magnetic nanoparticles at 300 Gauss and 305kHz. A preculture of C6 cells was performed before the 3D cells culture on the chip. The process of magnetic hyperthermia on the Chip was performed after administration of 20*μ*L of magnetic nanoparticles (10mgFe/mL) using the parameters that generated the specific absorption rate value. The efficacy of magnetic hyperthermia therapy was evaluated by using the cell viability test through the following fluorescence staining: calcein acetoxymethyl ester (492/513nm), for live cells, and ethidium homodimer-1 (526/619nm) for dead cells dyes.

**Results:**

Magnetic nanoparticles when submitted to the alternating magnetic field (300 Gauss and 305kHz) produced a mean value of the specific absorption rate of 115.4±6.0W/g. The 3D culture of C6 cells evaluated by light field microscopy imaging showed the proliferation and morphology of the cells prior to the application of magnetic hyperthermia therapy. Fluorescence images showed decreased viability of cultured cells in organ-on-a-Chip by 20% and 100% after 10 and 30 minutes of the magnetic hyperthermia therapy application respectively.

**Conclusion:**

The study showed that the therapeutic process of magnetic hyperthermia in the glioblastoma on-a-chip model was effective to produce the total cell lise after 30 minutes of therapy.

## INTRODUCTION

**Figure 1:**
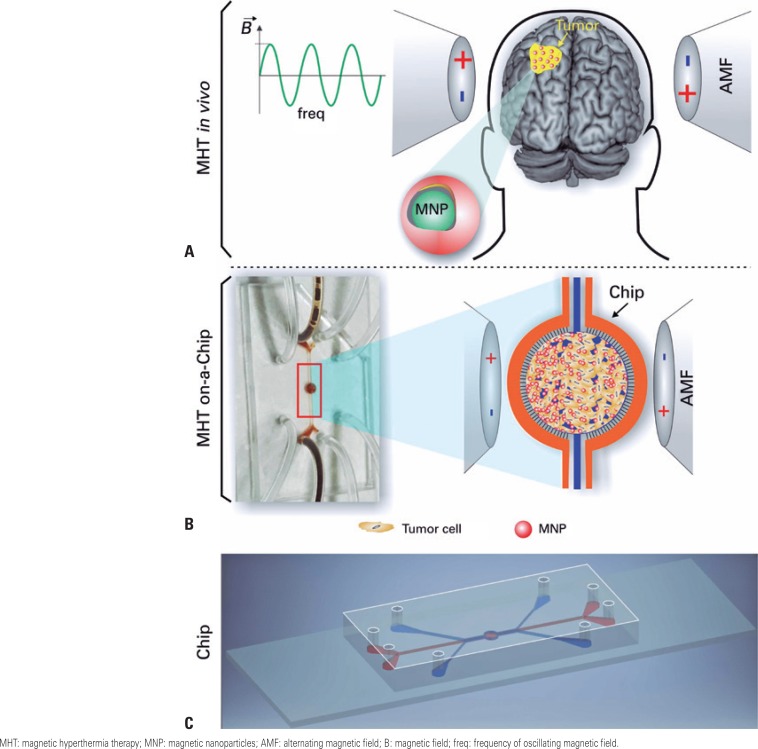
Magnetic hyperthermia therapy. (A) *In vivo* magnetic hyperthermia therapy: magnetic nanoparticles are injected locally in glioblastoma tumor and submitted to alternating magnetic field; (B) On-a-chip magnetic hyperthermia process: dispersed magnetic nanoparticles in aqueous medium are injectable in central cavity of the chip, becoming in contact with 3D cell culture of tumor cells and posteriorly submitted to alternating magnetic field. The red drawing in frame shows brown solution of magnetic nanoparticles filling the chip cavity; (C) Microfluidic device (chip)

In the last decade, nanotechnology and nanomaterials has led to advances and introduced a new area in medicine, the nanomedicine.^(^^1^^,^^2^^)^ As a result of the studies developed in this area, a number of products have been commercialized and used in clinical medicine,^(^^3^^,^^4^^)^ such as biocompatible nanoparticles applied both to therapy and to diagnosis of tumors.^(^^5^^)^ One of the techniques applied to treatment of tumors is hyperthermia that uses strategies based on radiofrequency waves, ultrasound and microwave, as well as laser light treatments associated with the use of nanoparticles with magnetic properties.^(^^6^^–^^8^^)^ Magnetic nanoparticles (MNP), when submitted to alternating magnetic field (AMF), generate heat by transforming magnetic energy into thermal energy used in treatment of tumors, such as shown in figure 1. The increase of temperature, called hyperthermia, by using MNP, corresponds to what is called magnetic hyperthermia therapy (MHT). The MHT has been investigated for treatment of glioblastoma (GBM) tumors because of the fact that these primary malignant brain tumors are more lethal and they present resistance to treatment of chemotherapy and radiotherapy, an event after surgical intervention. Still, although a number of therapeutic advances have been achieved, the development of new studies about treatment of GBM is scarce.^(^^9^^)^

Treatment of GBM, using the MHT technique,^(^^10^^,^^11^^)^ consist of administration of MNP in tumor tissue and heating of tumor cells to temperature between 41 to 43°C, by application of AMF, depending on magnetic field (B) and frequency of oscillation (freq) as shown in figure 1A.

Glioblastoma tumors studies with MHT technique have been emphasized because they increase patient's survival compared with other treatments, in addition to not present relevant collateral effects.^(^^12^^,^^13^^)^ The assessment of new therapeutic approach for treatment of GBM by using MHT technique was showed *in vitro* and murine models, however, this *in vitro* models did not respond clearly the reason why they are incapable of mimic tumor in an environment that the tumor is, and, in murine models, there are a difference between use xerographic or autologous models.^(^^14^^)^ However, in 3D cell culture model, which use technology of microfluidics devices (chip), cultivated tumor cells mimic the development of tumor tissue most near to reality,^(^^14^^)^ enabling, in this system, the assessment of therapeutic approaches, such as MHT, which does not occur in model *in vitro* 2D, which is not so efficient to visualize the behavior of cancer cells during coculture.^(^^15^^,^^16^^)^

By using organ-on-a-chip modality, it is possible to mimic tumors of GBM and apply therapy of MHT after administration of dispersed MNP in aqueous medium in cavities of chip, such as shown in figure 1B. There are some appropriated geometrics for development of GBM model on-a-chip, such as adequate channels for development of tumor tissue, GBM on-a-chip, such as adequate channels for development of tumor tissue, correct administration of drugs/nanoparticles, change of fluids for cell maintenance, among other (figure 1C). Other advantage of development of tumor tissue and assessment of therapy of MHT on-a-chip is the adequacy to guidelines of 3Rs (Replacement, Reduction, and Refinement) reducing the use of animals.

## OBJECTIVE

To evaluate efficiency of magnetic hyperthermia therapy in glioblastoma on-a-chip.

## METHODS

The study was carried out in an Experimental Research Center and Experimental and Surgical Training Center of the Teaching and Research Institute at *Hospital Israelita Albert Einstein*, Sao Paulo, Brazil.

### Magnetic nanoparticles

Dispersed MNP in aqueous medium forming one ferrofluid, available commercially as fluidMAG-Amine (Chemicell, Berlin, Germany), have used in therapy of MHT in GBM on-a-chip model. The MNP has nucleus with crystalline phase of magnetite (Fe_3_O_4_), being coated with aminosilane, which turns it biocompatible. The hydrodynamic diameter of MNP is 100nm with number of nanoparticles ~1.8×10^15^/g and density of ~1.25g/cm^3^.

### Description of magnetic hyperthermia equipment

Therapy of MHT in microfluidic device was applied using system of magnetic heating composed by: DM100 applicator (nB nanoScale Biomagnetics, Zaragoza, Spain) of adjustable magnetic field (50-300 Gauss) in a variety of modes of frequencies (305, 557, 715 and 874kHz); and controller module DMC1 (nB nanoScale Biomagnetics, Zaragoza, Spain), which enabled to conduct program of trials, monitoring of measures and analysis of results. The monitoring of temperature was taken using fiber optic temperature measurement sensors (Luxtron 3204, Luxtron Corporation, Northwestern Parkway, CA, USA). The system was controlled by software MaNiaC (nB nanoScale Biomagnétics, Zaragoza, Spain), which enabled program and data processing.

### Determination of specific absorption rate of magnetic nanoparticles of iron oxide

Determination of specific absorption rate (SAR) of MNP (10mgFe/mL) was conducted by submitting these to AMF (300 Gauss, 305kHz), registering ranging temperature over time that, for statistical purposes, four measurements were done. Calculations of SAR was done using MaNiaC software, using the relation

$$SAR\left(W/g\right)=\frac{m_{NP}c_{NP}+m_{1}c_{1}}{m_{NP}}\left(dT/dt\right)max,$$

in which *m*_*NP*_ is the mass of MNP, *m*_*1*_ the mass of water (1000kg/m^3^), *c*_*NP*_ the specific heat of magnetite (0.16kCal/kg°C), *c*_*1*_ the specific heat of water (1.0kCal/kg°C) and (*dT/dt*)*max* is maximal ranging of heating curve of MNP.

### Cell pre-culture of C6 lineage

In this study, we used C6 cells of glioma, a GBM lineage multiform of rats (Cell bank of Rio de Janeiro - BCRJ, code: 0057), which has the ability to form *in vivo* tumors and share a number of malignant characteristics with GBM human.^(^^17^^,^^18^^)^

These C6 cells were cultivated using RPMI (GIBCO^®^ Invitrogen Corporation, CA, USA), supplemented with 10% of fetal bovine serum (FBS) (GIBCO^®^ Invitrogen Corporation, CA, USA), 1% of penicillin- streptomycin (GIBCO^®^ Invitrogen Corporation, CA, USA) and 1% of L-glutamine (GIBCO^®^ Invitrogen Corporation, CA, USA) at 37°C (5% CO_2_), in bottles of cell culture of 75cm^2^ (Corning, USA). When achieve cell confluence of 85%, cells were trypsinized using 0.25% trypsin (GIBCO^®^ Ivitrogen Corporation, CA, USA), collected, centrifuged at 800rpm for 5 minutes, resuspended in culture medium in cell concentration of 10^7^cells/mL and keep refrigerated on ice bath.

### 3D cell culture of C6 cells on-a-chip

To mimic tumor tissue of GBM, we used microfluidic device from SynVivo Inc (Huntsville, AL, US). This chip was formed by two compartments, one central and the other external, separated by porous interface, aiming the culture of the C6 cells in 3D in central cavity, being prepared as described in SynVivo protocol.^(^^19^^)^ Basically, 15*μ*L of Matrigel^®^ (40mg/cm^2^) (EMD Milipore, Billerica MA) were injected in central compartment, using a syringe and sterile Tygon tubing (0.02″ ID × 0.06″ OD) SynVivo Inc, Huntsville, AL, US) and kept on the fridge at 5°C for a period of 2 hours. Culture medium non-supplemented RPMI was injected for washing compartment, and C6 cells (10^7^cells/mL), with flow rate of 2*μ*L/minute, using bomb 11 Elite Nanomite (Harvard Apparatus, Holliston, MA). External compartment was supplemented RPMI with 10% SFB to 5*μ*L/minute and maintained during all period of culture. The chip was placed in 5% carbon dioxide oven and 37°C for 3D cell culture of C6 cells, with change of culture mean of cavity every 4 hours, during 48 hours.

### Assay of magnetic hyperthermia process in glioblastoma on-a-chip

**Figure 2:**
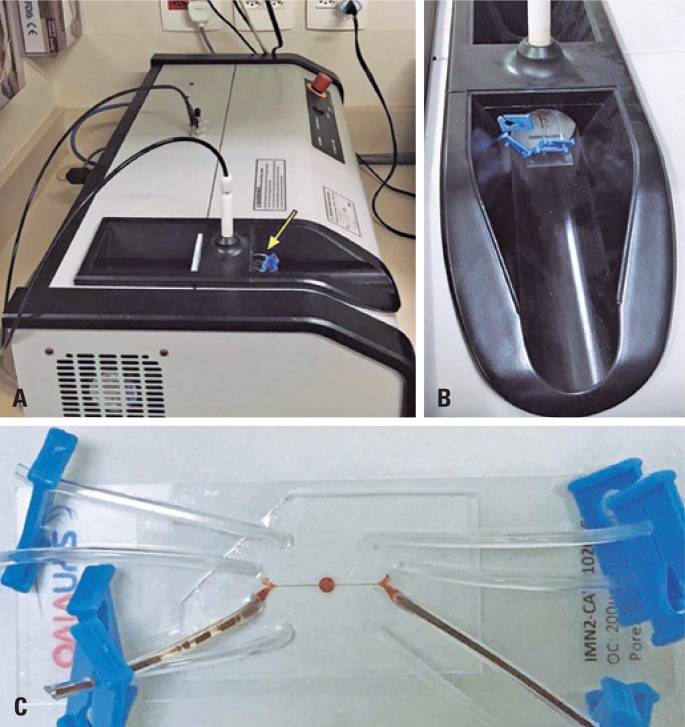
Application of magnetic hyperthermia to glioblastoma on-a-chip. (A) Equipment of magnetic hyperthermia and applicator of alternating magnetic field (yellow arrow indicating the chip localization); (B) Image showing the chip inserted in central region of coil that generate the alternating magnetic field; (C) Glioblastoma tumor on-a-chip for magnetic hyperthermia therapy

After 48 hours of 3D cell culture of C6 cells on-a-chip, we performed the MHT assay. For this reason, the chip was removed from the incubator, and central cavity was washed with RPMI (0%FBS); subsequently, we injected 20*μ*L of MNP, in concentration of 10mgFe/mL in the same local using infusion pump. The chip was taken up to the MHT equipment and placed on applicator, as shown in figure 2. The experiment was planned for application of AMF (300 Gauss, 305kHz) on chip for period of 30 minutes. Aiming to have temperature control in therapeutic range from 41 to 43°C, we used the amount colloidal suspension of MNP (200*μ*L contained in eppendorf in concentration of 10mgFe/mL) as referential sample that was submitted to same AMF together with chip and the temperature was monitored by fiber optic system.

### Evaluation of efficiency of magnetic hyperthermia process in glioblastoma on-a-chip

The assessment of efficiency of therapy of MHT in GBM on-a-chip was conducted using LIVE/DEAD^®^ kit (Molecular Probes, Eugene, Oregon, USA) of assay of viability and/or cell toxicity, through image of fluorescence, in which were used 4mM of Calcein acetoxymethyl ester (Ca-AM) and 12mM of ethidium homodimer-1 (EthD-1). The fluorescence of both staining occur to interact with live cells (for Ca-AM-excitation/emission: 492/513nm) or dead cells (for EthD-1 – excitation/emission: 526/619nm). Green fluorescence of acetoxymethyl ester indicates the activity of intracellular esterases of viable cells, and red fluorescence of EthD-1 indicates loss of integrity of plasmatic membrane. The analysis of cell viability before and after the MHT therapy in tumor cells on-a-chip was conducted by injecting 15*μ*L of solution formed by Ca-AM and EthD-1 in central cavity of chip and, subsequently, we registered fluorescence images, using inverted microscopy Nikon Eclipse Ti-E (Tokio, Japan). The counting of live (green) and dead (red) cells were done in two regions of organ-on-a-chip (region I and II, bifurcation of entrance of fluids and central cavity of chip, respectively). The experiment since the 3D cell cultivation up to assessment of cell viability was repeated three times.

## RESULTS

### Assessment of specific absortion rate of iron oxide magnetic nanoparticles

**Figure 3:**
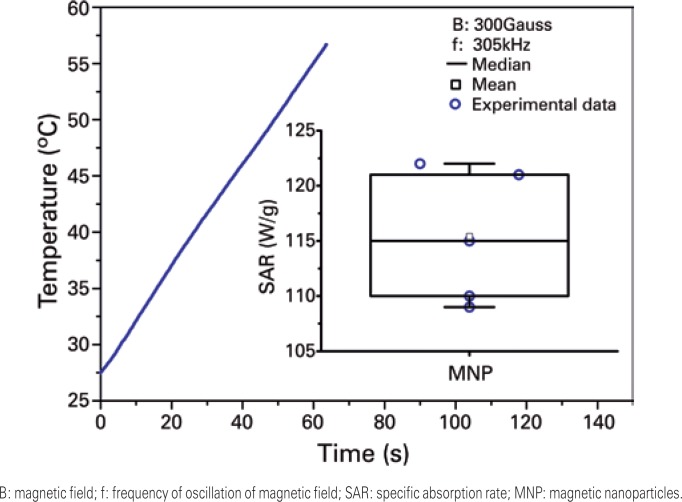
Heating curve of magnetic nanoparticles submitted to alternating magnetic field of 300 Gauss and frequency of oscillation of 305kHz. The inset shows the distribution of values of rate of specific absorption of magnetic nanoparticles

Capacity of heating of MNP was characterized for application in MHT. The heating curve of MNP is shown in figure 3, and indicates rapid increase of temperature in period of 60 seconds. The inset of figure 3 (box plot) shows the assessment of distribution of values of SAR with mean values of 115.4±6.0W/g.

### 3D cell culture of C6 cells on-a-chip

**Figure 4:**
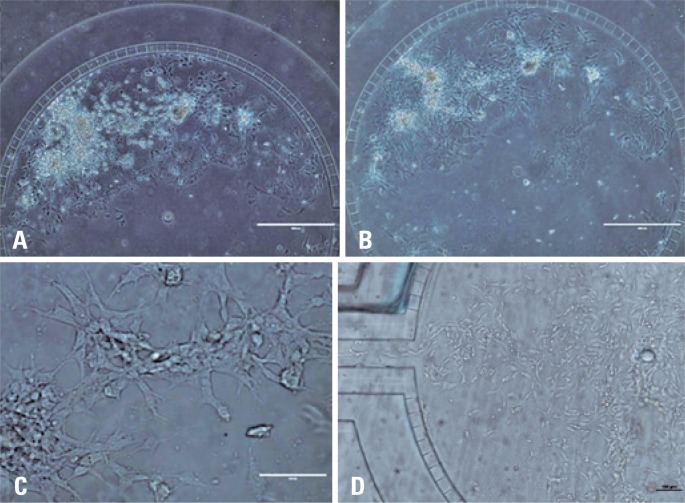
Microscopy images of clear field of 3D cell culture of C6 cells on-a-chip. Images of cells adhere to central cavity of chip after (A) 4 hours and (B) 48 hours of culture (4X); (C) C6 cell colony and their morphology (20X) and (D) Details of cell proliferation (4X)

3D cell culture of C6 cells was conducted in central cavity of chip and evaluated through images of microscopy of clear field. Figure 4, shows images of C6 cells in culture after 4 and 48 hours of sowing (Figures 4A and 4B), respectively. For this reason, morphology of C6 cells and their proliferation (Figures 4C and 4D) in central region of the chip.

Images of figure 4, we could observe the beginning of C6 cells growth in isolated regions forming islands, with cell proliferation within islands, beginning the 3D cell cultivation over the Matrigel^®^ structure mimicking the formation of GBM tumor tissue.

### Assessment of efficiency of magnetic hyperthermia therapy in glioblastoma on-a-chip

**Figure 5:**
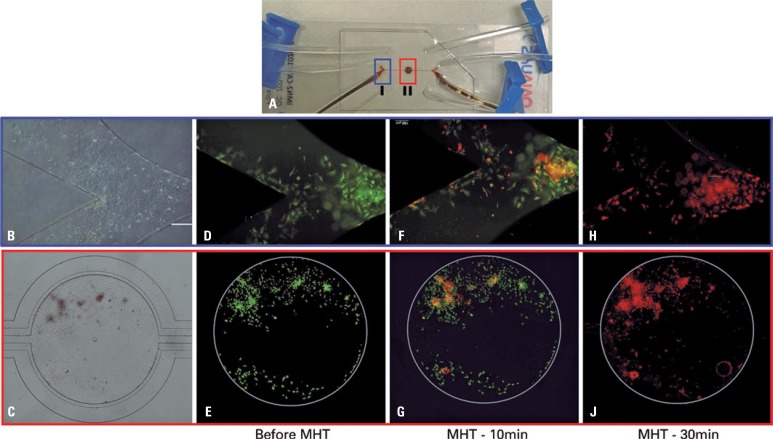
Viability of assay of C6 cells showing live cells stained with calcein acetoxymethyl ester (green) and dead cells stained with ethidium homodimer-1 (red). (A) In microfluidic device, the two regions of analysis are highlighted: blue, the region I (bifurcation of entrance of fluid on the chip) and, in red, the region II (central cavity of chip); (B and C) Images of microscopy of clear field, showing the cell cultivation in region I and II, respectively. (D and E) Images of fluorescence of live C6 cells (green) before magnetic hyperthermia therapy. (F and G) Images of live C6 cells of fluorescence images (green) and dead cells (red), after 10 minutes of magnetic hyperthermia on the chip. (H and J) Fluorescence images of dead C6 cells (red) in regions I and II of the analysis, after 30 minutes of magnetic hyperthermia therapy. All images presented are composed by overlap of images from analysis of each staining (Calcein acetoxymethyl ester and ethidium homodimer-1) and posterior subtraction of background

After growth of tumor tissue on the chip, we applied the therapy of MHT to conduct assay of viability of C6 cells, such as shown in figure 5. The microfluidic device in figure 5A shows regions of evaluation of cell viability indicated by frames in blue (region I, showing the bifurcation of fluid entrance) and red (region II, showing the central cavity). Figures 5B and 5C show images of microscopy of clear field of C6 cells cultivation in regions I and II, respectively. Figures 5D and 5E include images of fluorescence that correspond to live C6 cells reacting to Ca-AM before therapy of MHT in regions I and II of Chip, respectively. In figure 5F and 5G, we observed fluorescence image of region I and II with live C6 cells (green) and dead cells (red) that react to Ca-AM and EthD-1, respectively, after 10 minutes of therapy of MHT in GBM on-a-chip. In figure 5H and 5J, we observed by images of fluorescence of regions I and II, respectively, only dead C6 cells (red) that reacted with EthD-1 staining, after 30 minutes of MHT therapy.

Fluorescence assay of figure 5 showed that MNP with SAR value (115.4±6.0W/g) was adequate for heating of tumor tissue up to therapeutic temperature, when submitted to one AMF with magnetic field of 300 Gauss and frequency of 305kHz. Hyperthermia treatment showed a reduction of cell viability in 20%, after 10 minutes, and in 100% after 30 minutes of MHT, through the use of kit of cell viability (LIVE/DEAD^®^).

## DISCUSSION

Microfluidics have provided a large development in tissue engineering, aiming to understand biologic processes *in vitro* studies.^(^^20^^)^ The development of these microfluidic systems to mimic tumors are on use in a number of therapies, raising the interest of scientific community, in order to replace the use of murine models.^(^^21^^–^^23^^)^ One of these therapies is applied by hyperthermia treatments, such as MHT in tumors.

Studies of MHT in tumor cells using MNP show a large potential in treatment for tumors of GBM, however, because of variety of parameters of application of MHT and the use of different types of tumor cells, it has been difficult to evaluate which are the best parameters of this therapy in tumor treatment, as well as this constitutes a barrier for application of this modality as standard in treatment of GBM.^(^^24^^)^ This can be observed in review of Gupta et al.,^(^^25^^)^ which describe different parameters of applications of MHT in models of tumor *in vitro* and characteristics of MNP used. In the study by Hanini et al.,^(^^26^^)^ an evaluation was conducted of MHT in glioma cells (U87-MG) treated with MNP of γ-Fe_2_O_3_ coated with polyol, with diameter of 10nm, in concentration of 50*μ*gFe/mL, submitted to AMF with frequency of oscillation of 700kHz and magnetic field of 289.67Oe, with time of application of 60 minutes, keeping the therapeutic temperature of 42°C and showing reduction cell viability of 50%. In other study, we used cell of glioma T-9 and MNP of magnetite with diameter of 35nm in concentration of 7.2mgFe/mL, applying AMF with 118kHz and 383.72Oe, achieving cell lyse of 100% in 60 minutes.^(^^27^^)^ By using the same tumor cell used in our study, Gupta et al.,^(^^28^^)^ evaluated C6 glioma cells of rats and NIH3T3 fibroblast of mice, using MNP of Fe_2_O_3_ coated with steviosides, of 4.62nm of diameter, in concentration of 100*μ*gFe/mL, applying AMF with 405kHz and 168Oe for 30 minutes, achieving therapeutic temperature of 43°C, and showing a decrease in cell viability of 40% and, after 4 hours of coculture, an additional reduction of 34%. The enhancement of nanomaterial for this therapeutic approach has also been the focus of this study to obtain the best SAR that reflects in efficiency of MHT technique.

However, *in vivo* studies, such the one conducted by Jordan et al.,^(^^29^^)^ the efficacy of therapy of MHT was evaluated in brain tumor of Fisher's rats induced by RG-2 cells, with two types of MNP - one coated with aminosilane and other dextran. Results showed that application of AMF (100kHz and 225.72Oe) with MNP coated with aminosilane was more efficient in reduction of rate of cell proliferation than when coated with dextran. This study had adequate values of SAR, on the range of 10 to 100W/g – values considered typical of SAR for this type of application.^(^^30^^)^ In our study, the MNP of iron oxide (magnetite) were also coated with aminosilane and mean value of SAR of these MNP was 115.4±6.0W/g. Clinical studies published in the literature have also reported^(^^31^^–^^33^^)^ the used of this type of MNP coated with aminosilane, and parameters of application of MHT are similar to those used in our GBM study on-a-chip. These similar characteristic, also use the C6 tumor cells that mimic the human GBM, assist in transposition of data for human model, allowing better evaluation of altered therapeutic approach, such as the MHT, combined or not with other techniques in high-severity illness and low response to conventional treatments such as the GBM.

Currently, the organ on-a-chip model of GBM has been used to evaluate the ability to model progression of hyper cellular regions of GBM, observed in patients, and mimicking the obstruction of blood vessels, modeling the delivery of nutrients, and gradients of oxygen during the evolution of GBM;^(^^34^^)^ screening of high performance drugs and prolonged drug delivery;^(^^35^^–^^37^^)^ to evaluate vascular compartment that present a network of vessels in communication with solid 3D tumors mimicking microenvironment of tumor, including the knowledge as Enhanced Permeability and Retention (EPR),^(^^38^^)^ among others.

For this reason, the optimization of therapy of MHT in microfluidic device that mimic the characteristics of GBM is presented as potential application for translational in humans.

The models of GBM on-a-chip of our study provides basis for implementation of this method technique of MHT, aiming to evaluate its potential therapeutic in GBM, temporally, although our model has presented one limiting factor, which was the lack of vascular network associated with tumor tissue, but this must be implemented in future studies.

## CONCLUSION

Our study showed efficiency of magnetic hyperthermia therapy for treatment of glioblastoma on-a-chip with lise of all tumor cells after 30 minutes of magnetic hyperthermia using nanoparticles of iron oxide coated with aminosilane, which is used in clinical trials. In addition, specific absorption rate was often used in therapy assays of magnetic hyperthermia in tumors of human glioblastoma.
