# Supplementary material for: Magnetic hyperthermia therapy in glioblastoma tumor on-a-Chip model
Source: Einstein (Sao Paulo). 2019 Dec 20;18:eAO4954. doi: 10.31744/einstein_journal/2020AO4954 (PMC6924828; doi:10.31744/einstein_journal/2020AO4954)
Supplement: Translation

# Translation: Terapia de magneto-hipertermia no modelo de tumor de glioblastoma *on-a-Chip*

**Authors:** Javier Bustamante Mamani, Bruna Souto Marinho, Gabriel Nery de Albuquerque Rego, Mariana Penteado Nucci, Fernando Alvieri, Ricardo Silva dos Santos, João Victor Matias Ferreira, Fernando Anselmo de Oliveira, Lionel Fernel Gamarra

**Affiliations:** 1 Hospital Israelita Albert Einstein, São Paulo, SP, Brasil.; 2 Hospital das Clínicas, Faculdade de Medicina, Universidade de São Paulo, São Paulo, SP, Brasil.

**Keywords:** Glioblastoma/terapia, Hipertermia, Nanopartículas, Microfluídica, Células C6

## Abstract

**Objetivo:**

Avaliar a terapia de magneto-hipertermia em modelo de tumor de glioblastoma *on-a-Chip*.

**Métodos:**

As nanopartículas magnéticas recobertas com aminosilana foram utilizadas para a terapia da magneto-hipertermia, sendo avaliada a taxa de absorção específica das nanopartículas magnéticas em 300 Gauss e 305kHz. Uma pré-cultura de células C6 foi realizada e, seguidamente, foi feito o cultivo das células 3D no *chip*. O processo de magneto-hipertermia no *chip* foi realizado após administração de 20*μ*L de nanopartículas magnéticas (10mgFe/mL), utilizando os parâmetros que geraram o valor da taxa de absorção específica. A eficácia da terapia de magneto-hipertermia foi avaliada pela viabilidade celular por meio dos corantes fluorescentes acetoximetiléster de calceína (492/513nm), para células vivas, e etídio homodímero-1 (526/619nm), para células mortas.

**Resultados:**

As nanopartículas magnéticas, quando submetidas ao campo magnético alternado (300 Gauss e 305kHz), produziram um valor médio da taxa de absorção específica de 115,4±6,0W/g. A cultura 3D das células C6 avaliada por imagem de microscopia de campo claro mostrou a proliferação e a morfologia das células antes da aplicação da terapia de magneto-hipertermia. As imagens de fluorescência mostraram diminuição da viabilidade das células cultivadas no *organ-on-a-Chip* em 20% e 100% após 10 e 30 minutos, respectivamente, da aplicação da terapia de magneto-hipertermia.

**Conclusão:**

O processo terapêutico da magneto-hipertermia no modelo de tumor glioblastoma *on-a-chip* foi eficaz para produzir lise total das células após 30 minutos de terapia.

## INTRODUÇÃO

Na última década, a nanotecnologia e os nanomateriais deram lugar a uma nova área na medicina, a denominada “nanomedicina”.^(^^1^^,^^2^^)^ Como resultado das pesquisas desenvolvidas nesta área, diversos produtos já estão sendo comercializados e usados na clínica médica,^(^^3^^,^^4^^)^ como as nanopartículas biocompatíveis aplicadas tanto na terapia como no diagnóstico de tumores.^(^^5^^)^ Uma das técnicas aplicadas no tratamento de tumores é a terapia hipertérmica, que utiliza estratégias baseadas em ondas de radiofrequência, ultrassom e microndas, bem como tratamentos com laser associado ao uso de nanopartículas com propriedades magnéticas.^(^^6^^–^^8^^)^ As nanopartículas magnéticas (NPM), quando submetidas a um campo magnético alternado (CMA), geram aquecimento, mediante a transformação de energia magnética em energia térmica usada no tratamento de tumores, como mostrado na figura 1. O incremento da temperatura, denominando de hipertermia, usando NPM, corresponde ao que é chamado de terapia de magneto-hipertermia (MHT). A MHT tem sido alvo de pesquisas no tratamento de tumores de glioblastoma (GBM) devido ao fato de ser um dos tumores cerebrais malignos primários mais letais e que apresentam resistência ao tratamento de quimioterapia e radioterapia, mesmo após intervenção cirúrgica. Ainda que existam muitos avanços terapêuticos, o desenvolvimento de pesquisas sobre o tratamento do GBM é incipiente.^(^^9^^)^

**Figura 1:** Terapia de magneto-hipertermia. (A) Terapia da magneto-*hipertermia in vivo*: nanopartículas magnéticas são injetadas localmente no tumor de glioblastoma e submetidas ao campo magnético alternado; (B) Processo de magneto-hipertermia *on-a-chip*: nanopartículas magnéticas dispersas em meio aquoso são injetadas na cavidade central do chip, entrando em contato com a cultura 3D de células tumorais e submetidas posteriormente ao campo magnético alternado. O detalhe em quadro de cor vermelha mostra a solução das nanopartículas magnéticas em cor marrom preenchendo a cavidade do *chip*; (C) Dispositivo microfluídico (*chip*)

O tratamento do GBM, mediante a técnica de MHT,^(^^10^^,^^11^^)^ consiste na administração de NPM no tecido tumoral e no aquecimento das células tumorais a uma temperatura entre 41 a 43°C, mediante aplicação do CMA, dependente do campo magnético (B) e frequência de oscilação (freq) como é mostrado na figura 1A.

Estudos de GBM com a técnica da MHT vêm se destacando por apresentarem aumento da sobrevida do paciente quando comparado a outros tratamentos, além de não apresentar efeitos colaterais relevantes.^(^^12^^,^^13^^)^

A avaliação de novas abordagens terapêuticas visando ao tratamento do GBM por meio da técnica da MHT foi demonstrada nos modelos *in vitro* e murinos, mas os modelos *in vitro* não respondem de forma clara, porque não são capazes de mimetizar o tumor no ambiente no qual ele se encontra, e, nos modelos murinos, existe uma diferença entre usar modelos xenográficos ou autólogos.^(^^14^^)^ Entretanto, no modelo de cultura celular 3D, que utiliza a tecnologia dos dispositivos microfluídicos (*chip*), as células tumorais cultivadas mimetizam o desenvolvimento do tecido tumoral de maneira mais próxima do real,^(^^14^^)^ possibilitando, nesse sistema, a avaliação de abordagens terapêuticas, como da MHT, o que não acontece no modelo 2D *in vitro*, o qual não se mostra tão eficiente para visualizar o comportamento de células cancerígenas durante a cocultura.^(^^15^^,^^16^^)^

Com o uso da modalidade *organ-on-a-chip*, é possível mimetizar tumores de GBM e aplicação da terapia da MHT após a administração das NPM dispersas no meio aquoso nas cavidades do *chip*, como mostrado na figura 1B. Existem algumas geometrias apropriadas para o desenvolvimento do modelo de GBM *on-a-chip,* como canais adequados para o desenvolvimento do tecido tumoral, correta administração de drogas/nanopartículas, troca de fluidos para a manutenção celular, entre outras (Figura 1C). Outra vantagem de desenvolver o tecido tumoral e avaliar a terapia de MHT *on-a-chip* está na adequação às diretrizes dos 3Rs (Redução, refinamento e substituição - *replacement, reduction,* e *refinement*) reduzindo o uso de animais.

## OBJETIVO

Avaliar a eficiência da terapia de magneto-hipertermia em tumores de glioblastoma *on-a-chip*.

## MÉTODOS

O estudo foi realizado no Centro de Pesquisa Experimental e no Centro de Experimentação e Treinamento em Cirurgia do Instituto Israelita de Ensino e Pesquisa do Hospital Albert Einstein, São Paulo, Brasil.

### Nanopartículas magnéticas

As NPM dispersas em meio aquoso formando um ferrofluido, disponíveis comercialmente como *fluidMAG-Amine* (Chemicell, Berlin, Germany), foram usadas na terapia da MHT no modelo GBM *on-a-chip*. As NPM possuem núcleo com fase cristalina da magnetita (Fe_3_O_4_), sendo cobertas com aminosilana, que as torna biocompatíveis. O diâmetro hidrodinâmico das NPM é de 100nm, com número de nanopartículas ~1,8×10^15^/g e densidade de ~1,25g/cm^3^.

### Descrição do equipamento de magneto-hipertermia

A terapia de MHT no dispositivo microfluídico foi aplicada usando sistema de aquecimento magnético composto por: aplicador DM100 (nB nanoScale Biomagnétics, Zaragoza, Spain) de campo magnético ajustável (50-300 Gauss) em diversos modos de frequências (305, 557, 715 e 874kHz); e um módulo controlador DMC1 (nB nanoScale Biomagnétics, Zaragoza, Spain), que permitiu realizar a programação dos ensaios, monitorização das medições e análise dos resultados. A monitorização da temperatura foi realizada mediante sonda de temperatura de fibra ótica (Luxtron 3204, Luxtron Corporation, Northwestern Parkway, CA, USA). O sistema foi controlado pelo software MaNiaC (nB nanoScale Biomagnétics, Zaragoza, Spain), que facilitou a programação e o processamento de dados.

### Determinação da taxa de absorção específica das nanopartículas magnéticas de óxido de ferro

A determinação da taxa de absorção específica (SAR - *specific absorption rate*) das NPM (10mgFe/mL) foi realizada submetendo estas ao CMA (300 Gauss, 305kHz), registrando a variação da temperatura no tempo e que, para fins estatísticos, foram realizadas em quatro medidas. O cálculo da SAR foi realizado mediante o *software* MaNiaC, utilizando a relação

$$SAR\left(W\text{/}g\right)=\frac{m_{NP}c_{NP}+m_{1}c_{1}}{m_{NP}}\left(dT/dt\right)max,$$

onde *m_NP_* é a massa das NPM, *m_1_* massa da água (1000kg/m^3^), *c_NP_* o calor específico da magnetita (0,16kCal/kg°C), *c_1_* o calor específico da água (1,0kCal/kg°C) e (*dT/dt*)*max* é máxima variação da curva de aquecimento das NPM.

### Pré-cultura celular da linhagem C6

Foram utilizadas, neste estudo, células C6 de glioma, uma linhagem de GBM multiforme de ratos (Banco de Células de Rio de Janeiro – BCRJ, código: 0057), que tem a capacidade de formar tumores *in vivo* e compartilhar várias características malignas com o GBM humano.^(^^17^^,^^18^^)^ Estas células C6 foram cultivadas em meio RPMI (GIBCO^®^ Invitrogen Corporation, CA, USA), suplementado com 10% de soro fetal bovino (SFB) (GIBCO^®^ Invitrogen Corporation, CA, USA), 1% de penicilina-estreptomicina (GIBCO^®^ Invitrogen Corporation, CA, USA) e 1% de L-glutamina (GIBCO^®^ Invitrogen Corporation, CA, USA) a 37°C (5% CO_2_), em garrafas de cultura celular de 75cm^2^ (Corning, USA). Ao atingir confluência celular de 85%, as células foram tripsinizadas utilizando 0,25% tripsina (GIBCO^®^ Ivitrogen Corporation, CA, USA), coletadas, centrifugadas a 800rpm por 5 minutos, ressuspensas em meio de cultura na concentração celular de 10^7^ células/mL e mantidas resfriadas em banho de gelo.

### Cultura celular 3D das células C6 *on-a-chip*

Para mimetizar o tecido tumoral de GBM, foi utilizada o dispositivo microfluídico da SynVivo Inc (Huntsville, AL, US). Este *chip* era formado por dois compartimentos, um central e outro externo, separados por interface porosa, visando à cultura de células C6 em 3D na cavidade central, sendo preparado como descrito no protocolo da *SynVivo*.^(^^19^^)^ Basicamente, 15*μ*L de Matrigel^®^ (40mg/cm^2^) (EMD Milipore, Billerica MA) foram injetados no compartimento central, utilizando seringa e tubulação estéril de *Tygon* (0,02″ ID × 0,06″ OD) (SynVivo Inc, Huntsville, AL, US) e mantidos na geladeira a 5^o^C por um período de 2 horas. Meio de cultura RPMI não suplementado foi injetado para lavagem do compartimento, e as células C6 (10^7^celúlas/mL) foram semeadas na cavidade central, a uma vazão de 2*μ*L/minuto, utilizando bomba 11 Elite Nanomite (Harvard Apparatus, Holliston, MA). No compartimento externo, foi escoado RPMI suplementado com 10% SFB a 5*μ*L/minuto e mantido durante todo o período de cultura. O *chip* foi colocado em estufa de dióxido de carbono a 5% e 37°C para a cultura 3D das células C6, com troca do meio de cultura da cavidade a cada 4 horas, durante 48 horas.

### Ensaio do processo de magneto-hipertermia no glioblastoma *on-a-chip*

Após 48 horas de cultura 3D das células C6 *on-a-chip*, foi realizado o ensaio de MHT. Para tal finalidade, o *chip* foi retirado da estufa, e a cavidade central foi lavada com RPMI (0%SFB); seguidamente, foram injetados 20*μ*L de NPM, na concentração de 10mgFe/mL no mesmo local, utilizando a bomba de infusão. O *chip* foi levado ao equipamento de MHT e colocado no aplicador, como mostrado na figura 2. O experimento foi planejado para aplicação do CMA (300 Gauss, 305kHz) no *chip* por um período de 30 minutos. Com a finalidade de ter o controle da temperatura na faixa terapêutica de 41 a 43^o^C, foi utilizada quantidade da suspensão coloidal de NPM (200*μ*L contidos em um *eppendorf* na concentração de 10mgFe/mL) como amostra referencial, que foi submetida ao mesmo CMA, em conjunto com o *chip* e a temperatura monitorada mediante o sistema de fibra ótica.

**Figura 2:** Aplicação da magneto-hipertermia no glioblastoma *on-a-chip.* (A) Equipamento de magneto-hipertermia e o aplicador de campo magnético alternado (seta amarela indica a localização do *chip*); (B) Imagem mostrando o *chip* inserido na região central da bobina que gera o campo magnético alternado; (C) Tumor de glioblastoma *on-a-chip* para a terapia da magneto-hipertermia

### Avaliação da eficiência do processo de magneto-hipertermia no glioblastoma *on-a-chip*

A avaliação da eficiência da terapia de MHT no GBM *on-a-chip* foi realizada usando o *kit* LIVE/DEAD^®^ (Molecular Probes, Eugene, Oregon, USA) de ensaio de viabilidade e/ou toxicidade celular, mediante imagem de fluorescência, no qual foram usados 4mM de acetoximetiléster de calceína (Ca-AM) e 12mM de etídio homodímero-1 (EthD-1). A fluorescência de ambos os corantes ocorre ao interagir com células vivas (para Ca-AM – excitação/emissão: 492/513nm) ou mortas (para EthD-1 – excitação/emissão: 526/619nm). O verde fluorescente da acetoximetiléster indica a atividade de esterase intracelular de células viáveis, e o vermelho fluorescente do EthD-1 indica perda de integridade da membrana plasmática. A análise da viabilidade celular antes e após a terapia de MHT nas células tumorais *on-a-chip* foi realizada injetando 15*μ*L da solução formada por Ca-AM e EthD-1 na cavidade central do *chip* e, seguidamente, foram registradas imagens de fluorescência, utilizando um microscópio invertido Nikon Eclipse Ti-E (Tokio, Japan). As contagens de células vivas (verde) e mortas (vermelhas) foram feitas nas duas regiões do *organ-on-a-chip* (região I e II, a bifurcação da entrada dos fluídos e a cavidade central do *chip*, respectivamente). O experimento desde o cultivo celular 3D até a avaliação da viabilidade celular foi repetido três vezes.

## RESULTADOS

### Avaliação da taxa de absorção específica das nanopartículas magnéticas de óxido de ferro

A capacidade de aquecimento das NPM foi caracterizada para aplicação na terapia da MHT. A curva de aquecimento das NPM é mostrada na figura 3, indicando rápido incremento da temperatura em um período de 60 segundos. O *inset* da figura 3 (*box plot*) mostra a avalição da distribuição dos valores da SAR com valor médio de 115,4±6,0W/g.

**Figura 3:** Curva de aquecimento das nanopartículas magnéticas submetidas a um campo magnético alternado de 300 Gauss e frequência de oscilação de 305kHz. O *inset* mostra a distribuição dos valores da taxa de absorção específica das nanopartículas magnéticas

### Cultura celular 3D das células C6 *on-a-chip*

A cultura 3D das células C6 foi realizada na cavidade central do *chip* e avaliada mediante imagens de microscopia de campo claro. A figura 4, mostra imagens de células C6 em cultura após 4 e 48 horas da semeação (Figuras 4A e 4B), respectivamente, assim como a morfologia das células C6 e sua proliferação (Figuras 4C e 4D) respectivamente, na região central do *chip*.

**Figura 4:** Imagens de microscopia de campo claro da cultura celular 3D de células C6 *on-a-chip*. Imagem de células aderidas na cavidade central do chip após (A) 4 horas e (B) 48 horas de cultivo (4X); (C) Colônia de células C6 e sua morfologia (20X) e (D) Detalhes da proliferação celular (4X)

Nas imagens da figura 4, pode-se observar o início do crescimento das células C6 em regiões isoladas formando ilhas, com a proliferação celular nas ilhas, dando início ao cultivo celular em 3D sobre a estrutura de Matrigel^®^ mimetizando a formação de um tecido tumoral de GBM.

### Avaliação da eficiência da terapia da magneto-hipertermia no tumor de glioblastoma *on-a-chip*

Após crescimento do tecido tumoral no *chip*, foi aplicada a terapia de MHT para realização do ensaio de viabilidade das células C6, como mostrado na figura 5. O dispositivo microfluídico na figura 5A mostra as regiões de avaliação da viabilidade celular indicadas por quadros nas cores azul (região I, mostrando a bifurcação da entrada de fluídos) e vermelho (região II, mostrando a cavidade central). As figuras 5B e 5C mostram imagens de microscopia de campo claro do cultivo de células C6 nas regiões I e II, respectivamente. Nas figuras 5D e 5E, são apresentadas imagens de fluorescência que correspondem às células C6 vivas reagentes a Ca-AM antes da terapia de MHT, nas regiões I e II do *Chip*, respectivamente. Na figura 5F e 5G, observamos a imagem de fluorescência da região I e II com células C6 vivas (verde) e mortas (vermelho) que reagiram ao Ca-AM e EthD-1, respectivamente, após 10 minutos da terapia de MHT no GBM *on-a-chip*. Na figura 5H e 5J, verificamos, por meio das imagens de fluorescência das regiões I e II, respectivamente, somente as células C6 mortas (vermelho) que reagiram com o corante EthD-1, após 30 minutos de terapia de MHT.

**Figura 5:** Ensaio de viabilidade das células C6 mostrando células vivas coradas com acetoximetiléster de calceína (verde) e células mortas coradas com etídio homodímero-1 (vermelho). (A) No dispositivo microfluídico, estão ressaltadas as duas regiões de análise: na cor azul, a região I (a bifurcação da entrada de fluídos no *chip*) e, na cor vermelha, a região II (a cavidade central do *chip*); (B e C) Imagens de microscopia de campo claro, mostrando o cultivo de células nas regiões I e II, respectivamente. (D e E) Imagens de fluorescência das células C6 vivas (em verde) antes da terapia da magneto-hipertermia. (F e G) Imagens de fluorescência das células C6 vivas (em verde) e mortas (em vermelho), após 10 minutos da terapia da magneto-hipertermia no *chip.* (H e J) Imagens de fluorescência das células C6 mortas (em vermelho) nas regiões I e II de análise, após 30 minutos de terapia de magneto-hipertermia. Todas as imagens de fluorescência apresentadas são compostas da sobreposição das imagens resultantes da análise de cada corante fluorescente (acetoximetiléster de calceína e etídio homodímero-1) e subtração posterior do *background*

Os ensaios de fluorescência da figura 5 mostraram que NPM com valor de SAR (115,4±6,0W/g) foram adequadas para o aquecimento do tecido tumoral até a temperatura terapêutica, quando submetidas a um CMA com campo magnético de 300 Gauss e frequência de 305kHz. O tratamento hipertérmico mostrou redução da viabilidade celular em 20%, após 10 minutos, e em 100% após 30 minutos de MHT, mediante o uso do *kit* de viabilidade celular (LIVE/DEAD^®^).

## DISCUSSÃO

A microfluídica tem proporcionado grande desenvolvimento na engenharia de tecidos, visando ao entendimento de processos biológicos em estudos *in vitro*.^(^^20^^)^ O desenvolvimento destes sistemas microfluídicos para mimetizar tumores está sendo utilizado em diversas terapias, despertando o interesse na comunidade científica, afim de substituir o uso de modelos murinos.^(^^21^^–^^23^^)^ Uma destas terapias é a aplicação de tratamentos hipertérmicos, como o MHT em tumores.

Estudos de MHT em células tumorais utilizando NPM mostram grande potencial no tratamento para tumores de GBM, porém, devido à variedade de parâmetros da aplicação de MHT e ao uso de diferentes tipos de células tumorais, tem sido difícil a avaliação de quais são os melhores parâmetros desta terapêutica no tratamento tumoral, bem como configura-se um obstáculo para aplicação desta modalidade como padrão no tratamento de GBM.^(^^24^^)^ Isto pode ser verificado na revisão de Gupta et al.,^(^^25^^)^ que descreveram os diferentes parâmetros de aplicação do MHT em modelos de tumor *in vitro* e as características da NPM utilizadas. No estudo de Hanini et al.,^(^^26^^)^ foi realizada a avaliação do MHT em células de glioma (U87-MG) tratadas com NPM de γ-Fe_2_O_3_ recobertas com poliol, com diâmetro de 10nm, na concentração de 50*μ*gFe/mL, submetidas ao CMA com frequência de oscilação de 700kHz e campo magnético de 289,67Oe, com tempo de aplicação de 60 minutos mantendo-se a uma temperatura terapêutica de 42°C e mostrando diminuição na viabilidade celular de 50%. Em outro estudo, foram utlizadas células de glioma T-9 e NPM à base de magnetita com diâmetro de 35nm na concentração de 7,2mgFe/mL, aplicando CMA com 118kHz e 383,72Oe, alcançando lise celular de 100%, em 60 mimutos.^(^^27^^)^ Já com a mesma célula tumoral utilizada em nosso estudo, Gupta et al.,^(^^28^^)^ avaliaram as células de glioma C6 de rato e fibroblasto de camundongo NIH3T3, utilizando NPM de Fe_2_O_3_ revestidas com esteviosídeo, de 4,62nm de diâmetro, na concentração de 100*μ*gFe/mL, aplicando CMA com 405kHz e 168Oe durante 30 minutos, alcançando temperatura terapêutica de 43°C, e mostrando decréscimo na viabilidade celular de 40% e, após 4 horas de cocultura, diminuição adicional de 34%. O aprimoramento dos nanomateriais para esta abordagem terapêutica também tem sido foco de estudo para a obtenção da melhor SAR, que reflete na eficiência da técnica de MHT.

Já em estudos *in vivo*, como de Jordan et al.,^(^^29^^)^ foi avaliada a eficácia da terapia da MHT, em tumor cerebral de ratos Fisher induzido com células RG-2, com dois tipos de NPM, uma recoberta com aminosilana e outra com dextrana, mostrando que a aplicação do CMA (100kHz e 225,72Oe) com NPM recoberta com aminosilana foi mais eficiente na diminuição da taxa de proliferação celular do que quando revestidas com dextrana. Este estudo apresentou valores de SAR adequada, na faixa de 10 a 100W/g – valores considerados típicos de SAR para este tipo de aplicação.^(^^30^^)^ Em nosso estudo, as NPM de óxido de ferro (magnetita) também foram recobertas com aminosilana e o valor médio de SAR destas NPM foi de 115,4±6,0W/g. Estudos clínicos reportados na literatura^(^^31^^–^^33^^)^ também têm usado este tipo de NPM recoberto com aminosilana, e os parâmetros de aplicação de MHT são semelhantes aos usados em nosso estudo de GBM *on-a-chip*. Estas características similares, como também a utilização de células tumorais C6 que mimetizam o GBM humano, auxiliam na transposição dos dados para o modelo humano, permitindo melhor avaliação de abordagens terapêuticas alternativas, como o MHT, combinadas ou não com outras técnicas em doenças com alta severidade e baixa resposta aos tratamentos convencionais como são as GBM.

Atualmente, o modelo *organ on-a-chip* de GBM tem sido utilizado para avaliar a capacidade de modelar a progressão de regiões hipercelulares do GBM, observada em pacientes, imitando a obstrução de vasos sanguíneos e modelando a entrega de nutrientes e gradientes de oxigênio durante a evolução do GBM;^(^^34^^)^ triagem de drogas de alto rendimento e liberação prolongada de drogas;^(^^35^^–^^37^^)^ avaliar o compartimento vascular que apresenta uma rede de vasos em comunicação com tumores sólidos 3D imitando microambiente do tumor, incluindo o conhecido como efeito *Enhanced Permeability and Retention* (EPR),^(^^38^^)^ entre outros. Portanto, a optimização da terapia da MHT em um dispositivo microfluídico que mimetize as características do GBM se apresenta com potencial aplicação para o translacional em humanos.

O modelo de GBM *on-a-chip* do presente trabalho proporcionou bases para a implementação da metodologia da técnica de MHT, visando avaliar seu potencial terapêutico em GBM temporalmente, embora nosso modelo tenha apresentado um fator limitante, que foi a falta da rede vascular associada ao tecido tumoral, mas que deve ser implementada em trabalhos futuros.

## CONCLUSÃO

O presente estudo mostrou eficiência da terapia de magneto-hipertermia no tratamento de tumor de glioblastoma *on-a-chip*, com lise de todas as células tumorais após 30 minutos de terapia de magneto-hipertermia, usando nanopartículas de óxido de ferro recobertas com aminosilana (usadas em ensaios clínicos). Também, o valor de taxa de absorção específica foi o tipicamente usado em ensaios de terapia de magneto-hipertermia em tumores de glioblastoma humano.
